# Adipose Tissue Mast Cells Promote Human Adipose Beiging in Response to Cold

**DOI:** 10.1038/s41598-019-45136-9

**Published:** 2019-06-17

**Authors:** Brian S. Finlin, Amy L. Confides, Beibei Zhu, Mary C. Boulanger, Hasiyet Memetimin, Kyle W. Taylor, Zachary R. Johnson, Philip M. Westgate, Esther E. Dupont-Versteegden, Philip A. Kern

**Affiliations:** 1 The Department of Internal Medicine, Division of Endocrinology, and the Barnstable Brown Diabetes and Obesity Center, University of Kentucky, Lexington, KY 40536 United States; 2 Department of Rehabilitation Sciences, College of Health Sciences and Center for Muscle Biology, University of Kentucky, Lexington, KY 40536 United States; 3 College of Public Health, University of Kentucky, Lexington, KY 40536 United States

**Keywords:** Diseases, Endocrine system and metabolic diseases

## Abstract

In a recent study, repeated cold application induced beiging in subcutaneous white adipose tissue (SC WAT) of humans independent of body mass index. To identify factors that promote or inhibit beiging, we performed multiplex analysis of gene expression with the Nanostring nCounter system (the probe set contained genes for specific immune cell markers, cytokines, and chemokines) on the SC WAT from lean subjects. Multiple correlations analysis identified mast cell tryptase and CCL26, a chemokine for mast cells, as genes whose change correlated positively with the change in UCP1 in SC WAT, leading to the hypothesis that mast cells promote SC WAT beiging in response to cold. We quantified mast cell recruitment into SC WAT and degranulation. Mast cells increased in number in SC WAT in lean subjects, and there was an increase in the number of degranulated mast cells in both lean subjects and subjects with obesity. We determined that norepinephrine stimulated mast cell degranulation and histamine release *in vitro*. In conclusion, cold stimulated adipose tissue mast cell recruitment in lean subjects and mast cell degranulation in SC WAT of all research participants independent of baseline body mass index, suggesting that mast cells promote adipose beiging through the release of histamine or other products.

## Introduction

Subcutaneous white adipose tissue (SC WAT) of adult humans is usually composed of adipocytes that function to store lipid in a single large lipid droplet. In response to cold or β-adrenergic receptor agonists, SC WAT undergoes a process called beiging^1^. Beige adipose has increased abundance of uncoupling protein 1 (UCP1), which promotes thermogenesis by uncoupling the electron transport chain in mitochondria. In addition to thermogenesis, beige adipocytes are associated with improved glucose and lipid metabolism in rodents^2,3^. Thus, understanding mechanisms that regulate beiging is an important goal towards identifying strategies that can be therapeutically exploited to improve glucose and lipid homeostasis. Beiging has been extensively studied in rodents, and more recently, beiging of SC WAT has been demonstrated in humans^4–12^. Our recent study indicates that beige adipose can be induced in lean humans or humans with obesity by cold or by treatment of subjects with obesity with the β3 agonist mirabegron^6^.

In addition to β-adrenergic signaling and the sympathetic nervous system, the immune system has been shown to modify adipose beiging (recently reviewed^13^). Studies in mice have implicated macrophages, eosinophils, type 2 innate lymphoid cells, and iNKT cells in SC WAT beiging^13^. In addition, our recent study in humans implicated mast cells in the seasonal regulation of UCP1^7^. We found that mast cells function as cold sensors that release histamine, which stimulates lipolysis and UCP1 induction in adipocytes^7^. These immune cells interact in complicated ways with each other, with adipocytes, and with nerves to influence catecholamine levels, innervation, and the production of numerous factors that promote adipose beiging^13–22^. Alternatively, macrophage retention in adipose tissue has been shown in mice to inhibit beiging^23^, and *in vitro* studies have shown that macrophage conditioned medium and inflammatory cytokines inhibit UCP1 expression in adipocytes^5,23–26^.

We have recently shown that an acute localized cold exposure on one leg induced SC WAT beiging in humans^6^. Cold increased UCP1 and TMEM26 to the same extent in SC WAT from both legs, suggesting that SNS activation induces beiging rather than a localized decrease in SC WAT temperature^6^. Here, in that same cohort of lean subjects, we measured SC WAT gene expression of immune cell markers, cytokines, chemokines, and proteins involved in adipocyte function and dysfunction to gain further mechanistic insight into the beiging response in humans. We performed multiple correlations analysis to identify genes whose change in expression correlated with the change in UCP1 protein expression. In the cold treated leg, this analysis identified tryptase, an enzyme specifically expressed by mast cells, and CCL26, a chemokine for CCR3 receptor expressing cells, including mast cells, basophils, and eosinophils^27–29^, implicating mast cells in the induction of UCP1 expression by cold. We hypothesized that mast cell degranulation is involved in SC WAT beiging in response to cold, and therefore characterized mast cell recruitment and degranulation in SC WAT in response to cold and performed *in vitro* studies on mast cell degranulation in response to cold and norepinephrine.

## Research Design and Methods

### Human subjects and study design

The baseline characteristics and additional details about the research participants have been described elsewhere^6^. In brief, subjects were recruited from the Lexington, KY area in the summer (June 1 and September 15; mean temperature 20–24 °C). Baseline biopsies of thigh adipose were performed, the subjects then applied an icepack to one leg for 30 minutes each day for 10 consecutive days, and thigh biopsies were performed on the cold treated leg and the contralateral leg^6^. All subjects gave informed consent, and the protocols were approved by the Institutional Review Board at the University of Kentucky. All experiments were performed in accordance with relevant guidelines and regulations. The Clinicaltrials.gov registration identifier is NCT02596776 (date of registration: 04/11/2015).

### mRNA quantification

We used the Nanostring ncounter multiplex system to measure the expression of 130 genes and six housekeeping genes in purified RNA from SC WAT. The genes in the code set are described in Table S1 and reference^30^. βAR receptor expression was determined by real-time RT PCR as described^7^. The primer sequences are in Table S2.

### Immunohistochemistry

Mast cells were identified in SC WAT using mouse anti-tryptase (#sc-33676, Santa Cruz Biotechnology Inc, Dallas, TX). Sections were deparaffinized, subjected to antigen retrieval, blocked with 5% normal goat serum followed with a Streptavidin/Biotin block (# SP-2002, Vector Labs, Burlingame, CA), and then incubated consecutively with anti-tryptase primary antibody overnight. Samples were rinsed and incubated with biotinylated goat anti-mouse antibody (# 1-065-003, Jackson ImmunoResearch, West Grove, PA), strepavidin-HRP (#S911, Life Technologies, Carlsbad, CA), and then AlexaFluor 594 tyramide reagent (#B40957, Invitrogen, Carlsbad, CA). The slides were cover slipped using vectashield with DAPI (Vector Labs). Mast cells were counted in the non-fibrotic areas of the adipose tissue using images captured with a Zeiss AxioImager MI upright fluorescent microscope (Zeiss, Gottingen, Germany), and analysis was performed using Zen software (Zeiss). Degranulated mast cells were defined as having irregular shape with jagged edges and visible tryptase-filled vacuoles surrounding the cell. Capillary and vessel density was determined by staining with lectin-TRITC (#L4889, Sigma-Aldrich). Sections were prepared as above and incubated with lectin-TRITC for 2 hours followed by 4%PFA post fixation, and then cover slipped with vectashield with DAPI. Capillaries were counted as structures between 5 and 10 microns and vessels were above 10 microns.

### Histamine release

TIB64 cells (P815, ATCC, Manassas, VA) were grown at 37 °C in DMEM (#11885-092; Thermo Fisher Scientific, Grand Island, NY) with10% fetal bovine serum (FBS; #101; Tissue Culture Biologicals, Tulare, CA). One mL of TIB64 cells at a concentration of 1 × 10^6^ cells/mL was transferred to a centrifuge tube, spun down to remove growth medium, and resuspended in 2% FBS-DMEM medium warmed to the indicated temperature; the medium contained 100 nM norepinephrine as indicated. The cells were incubated at 37 °C or 32 °C for the indicated times and medium was harvested by centrifugation. Histamine was detected with a histamine EIA kit (Cayman Chemical, Ann Arbor, MI) following the manufacturer’s protocol.

### Statistics

Paired student’s t tests were conducted in Graphpad Prism version 7.0. The change in UCP1 protein determined by immunohistochemistry (post-pre) was determined from our previous study^6^. Histamine release was analyzed by one-way analysis of variance to determine group differences at both 120 and 240 minutes. Furthermore, pairwise comparisons were made based on Fisher’s least significant difference approach. Repeated measures multivariate analysis of variance (RM MANOVA) was performed as described^6^ to analyze mast cell recruitment and degranulation. Normality was assessed via the use of Q-Q plots. SAS version 9.4 was utilized for these analyses.

## Results

### Repeated cold exposure changes immune cell and angiogenic gene expression in SC WAT

We have recently characterized beiging of human SC WAT in response to cold^6^. The experimental design of that study was to biopsy SC WAT of one leg (baseline), apply an ice pack to that leg for 30 minutes a day for 10 days, and then perform SC WAT biopsies on both legs^6^. This design allowed us to address direct effects of cold and effects on sympathetic nervous system activation by cold (contralateral leg) on beiging. We observed equivalent beiging after cold exposure in SC WAT from both legs in 26 subjects with a wide range of body mass index (BMI), and notably, neither baseline body mass index nor insulin sensitivity affected the beiging response^6^. To gain a better understanding of human adipose beiging, we analyzed the mRNA expression of genes involved in adipocyte function, lipid metabolism, immune cell markers, chemokines, and inflammation (Table S1) in SC WAT obtained from lean subjects. As shown in Table 1, there was a significant decrease in CD68, a pan macrophage marker, and a significant increase in MRC2, a marker of alternative macrophage activation, in the cold treated leg. Cold decreased the gene expression of MMP9 and increased TIMP2 and thrombospondin 2. These gene changes suggest that macrophages and tissue remodeling are potentially involved in adipose tissue beiging, and this was further evaluated by multiple correlation analysis as described below. Finally, cold increased PPAR delta expression, which is involved in lipid catabolism.

**Table 1:** Gene Expression Changed in SC WAT of the Cold Treated Leg in lean research participants.

Gene Symbol^a^	Name	Pre	Post	Fold Change Post/Pre	P
ANGPT2	Angiopoietin 2	128 ± 9	181 ± 21	1.42	0.003
F3	Tissue Factor	737 ± 45	810 ± 49	1.10	0.011
MMP9	Matrix metallopeptidase 9	503 ± 134	262 ± 69	0.52	0.017
CD68	Cluster of Differentiation 68	1251 ± 185	843 ± 99	0.67	0.017
UCP2	uncoupling protein 2	816 ± 64	687 ± 69	0.84	0.017
PPARD	Peroxisome proliferator-activated receptor delta	44 ± 2	54 ± 3	1.25	0.017
MRC2	mannose receptor C type 2	513 ± 53	655 ± 78	1.28	0.031
FBXO31	F-box only protein 31	87 ± 5	105 ± 6	1.22	0.037
TIMP2	Tissue inhibitor of metalloproteinases 2	3054 ± 191	3514 ± 259	1.15	0.037
THBS2	Thrombospondin 2	505 ± 45	652 ± 76	1.29	0.038
NFE2L2	Nuclear factor (erythroid-derived 2)-like 2	958 ± 24	1055 ± 37	1.10	0.048

^a^SC WAT was isolated before and after cold treatment and gene expression determined with the Nanostring nCounter system as described in research design and methods. The data are presented as means (nCounter counts) ± the standard error of the mean (n = 12; two-tailed, paired student’s t-test).

We also analyzed gene expression in the contralateral leg since there was an increase in UCP1 in SC WAT that was similar to the cold-treated leg^6^. As shown in Table 2, different genes were changed in SC WAT of the contralateral leg in comparison to the cold-treated leg. Strikingly, the five genes with the lowest P values were all related to the regulation of angiogenesis including angiopoietin-2, which was also upregulated in the cold treated leg (Table 1). The expression of VEGFA was increased, and VEGFA has been found to stimulate adipose tissue beiging^31,32^. Because of these changes in angiogenic genes, we examined the vascularity (capillary density and larger blood vessels) the SC WAT from the cold-treated and contralateral legs of five random subjects, but did not find significant changes (Fig. S1). Other genes changed included tryptase, a marker of mast cells, and CCL26, which is chemotactic for CCR3 expressing cells such as basophils, mast cells, and eosinophils^27–29^. This finding was of interest since we recently defined a role for mast cells in seasonal induction of UCP1 mRNA^7^.

**Table 2:** Gene Expression Changed in SC WAT of the Contralateral Leg in lean research participants.

Gene Symbol^a^	Name	Pre	Post	Fold Change Post/Pre	P
VEGFA	Vascular endothelial growth factor A	1312 ± 78	1548 ± 82	1.18	0.006
ANGPT2	Angiopoietin 2	128 ± 9	163 ± 15	1.27	0.007
ANGPT4	Angiopoietin 4	14 ± 1	11 ± 1	0.75	0.009
ANGPT1	Angiopoietin 1	932 ± 84	826 ± 99	0.89	0.014
ANGPTL4	Angiopoietin Like 4	2063 ± 157	1585 ± 177	0.77	0.024
FNDC5	Fibronectin type III domain-containing protein 5	26 ± 3	21 ± 2	0.80	0.034
TPSAB1	Tryptase	2562 ± 318	3172 ± 398	1.24	0.034
IL18	Interleukin-18	80 ± 9	98 ± 10	1.23	0.047
CCL26	Chemokine (C-C motif) ligand 26	13 ± 2	17 ± 2	1.31	0.048
TNFRSF12A	TWEAK receptor	17 ± 2	14 ± 2	0.84	0.050

^a^SC WAT was isolated before and after Cold treatment and gene expression determined with the Nanostring nCounter system as described in research design and methods. The data are presented as means (nCounter Counts) ± the standard error of the mean (n = 12; two-tailed, paired student’s t-test).

**Figure 1:**
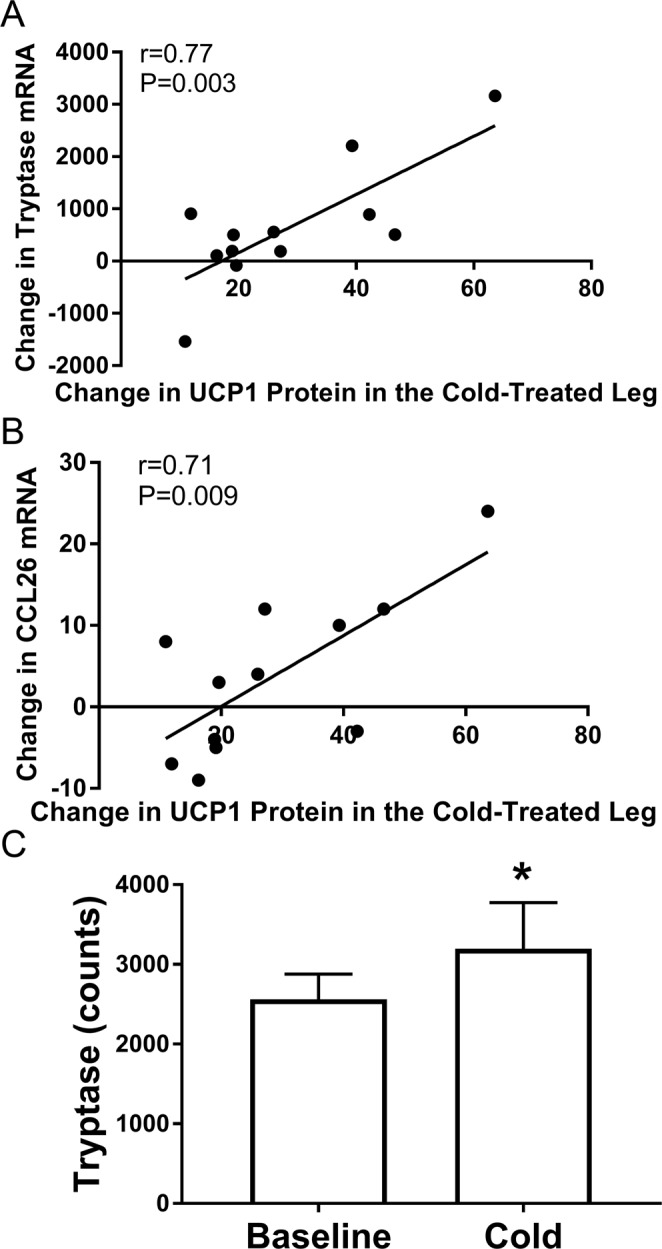
Correlations of changes in gene expression with changes in UCP1. The change in UCP1 protein expression in SC WAT by acute cold treatment (post – pre) was calculated for the cold treated and contralateral legs using our previously published results^6^. The change in UCP1 protein is plotted versus the change in gene expression of genes identified by multiple correlations analysis. (**A,B**) The change of tryptase or CCL26 versus the change in UCP1 protein the cold leg are shown. The data were analyzed by linear regression analysis, and Pearson correlation coefficients (r) and P values are shown (n = 12). (**C**) The quantification of tryptase gene expression at baseline and after cold in SC WAT of the cold treated leg is shown. Data represent means ± SEM (n = 12). *P < 0.05 (Wilcoxon matched-pairs signed rank test).

The analysis of gene expression above revealed numerous changes that may be related to the induction of UCP1 protein. We performed a multiple correlations analysis to identify changes in gene expression that predict the increase in UCP1 protein expression in SC WAT of the cold treated leg (Table 3) using the data on UCP1 protein expression that we previously determined^6^. This analysis identified the change in tryptase and CCL26 gene expression as positively correlated to the change in UCP1 protein expression (Table 3), suggesting mast cells are involved in the induction of UCP1 by cold. Figure 1A shows the positive correlation between the change in tryptase and the change in UCP1 expression in SC WAT of the cold treated leg, and Fig. 1B shows the result for CCL26. Tryptase was not found as a significantly increased gene in Table 1. Closer examination of tryptase gene expression in the cold treated leg revealed that the gene expression is not normally distributed at baseline and that tryptase gene expression significantly increases in the cold treated leg if non parametric analysis is used (Fig. 1C). We also investigated the relationship between tryptase and UCP1 in SC WAT of the contralateral leg and found a trend between the change in tryptase and the change in UCP1 protein expression (Fig. S2). Together, these findings suggest that cold stimulates adipose tissue mast cells, and this contributes to UCP1 induction. We also performed the multiplex analysis of gene expression in research participants with obesity and found that tryptase expression did not change significantly in either the cold treated or contralateral leg. Overall, the response of subjects with obesity was different and will be reported in the future. Notably, tryptase mRNA expression was not significantly altered by cold in the research participants with obesity.

**Table 3:** Multiple Correlations Analysis of Change in Gene Expression with Change in SC WAT UCP1 protein of the Cold Treated Leg.

Gene Symbol^a^	Name	r	P
**Positive correlation**			
TPSAB1	Tryptase	0.77	0.0033
CCL26	Chemokine (C-C motif) ligand 26	0.71	0.0092
**Negative correlation**			
ANGPTL4	Angiopoietin Like 4	−0.80	0.0016
TNFRSF12A	TWEAK receptor	−0.63	0.0324
CIDEA	cell death-inducing DNA fragmentation factor alpha-like effector A	−0.58	0.0464
ADIPOR2	Adiponectin receptor 2	−0.58	0.0467

^a^SC WAT was isolated before and after cold treatment^6^ and gene expression determined with the Nanostring nCounter system as described in research design and methods. We performed multiple correlations analysis to identify changes in gene expression that predicted the change in UCP1 protein expression as described in research design and methods. The data used to calculate the change in UCP1 were reported in^6^. Pearson correlation coefficients (r) and P values are given.

### SC WAT mast cells increase in number in lean subjects and degranulate in response to cold

**Figure 2:**
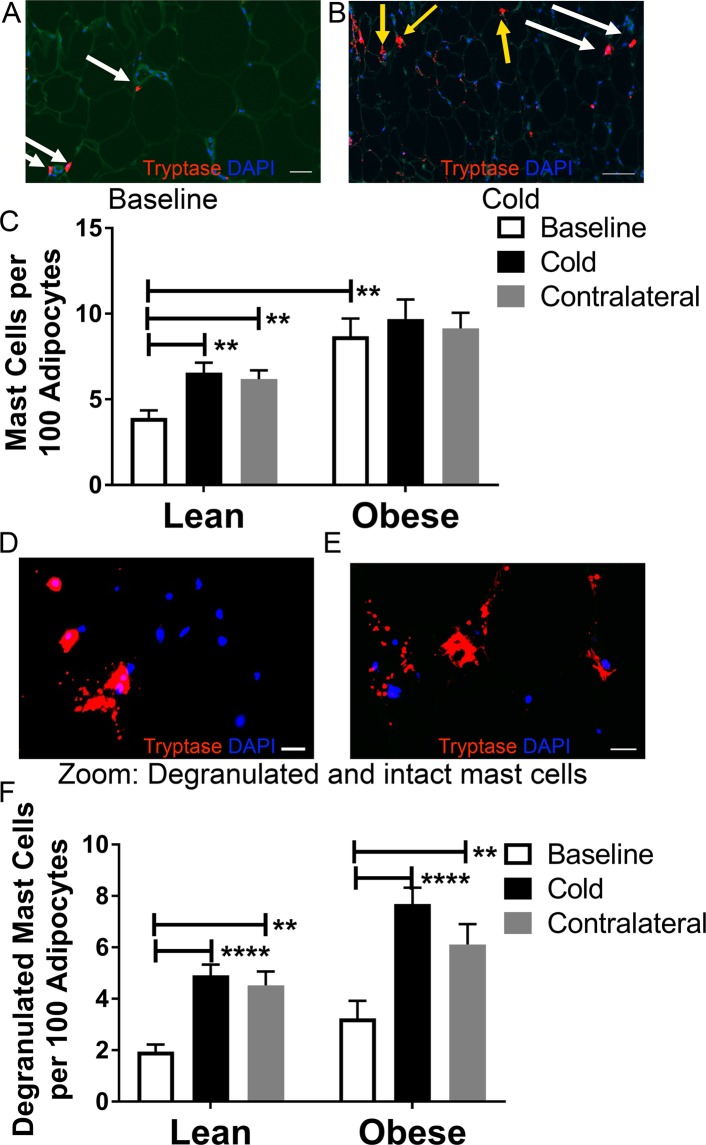
Mast cell density and degranulation in SC WAT of research participants in response to acute cold treatment. (**A,B**) Tryptase staining of SC WAT at baseline and after cold (scale bar for **C**: 50 μm; scale bar for **D**: 100 μm). White arrows point to intact mast cells and yellow arrows point to mast cells with diffuse, punctate staining. (**C**) Quantification of mast cell density in lean subjects and subjects with obesity at baseline and in SC WAT from the cold and contralateral legs after 10 days of acute cold exposure. (**D,E**) Higher magnification images demonstrating degranulated and intact mast cells (scale bar: 20 μm). (**F**) Quantification of degranulated mast cells in lean subjects and subjects with obesity at baseline and in SC WAT from the cold and contralateral legs after 10 days of acute cold exposure. Data represent means ± SEM. Data were analyzed by RM MANOVA as described in research design and methods. **P < 0.01; ****P < 0.0001 (lean n = 17; obese n = 8).

Cold could affect mast cell recruitment, stimulate mast cell degranulation, or affect both. We therefore investigated whether cold affects SC WAT mast cells in both the cold treated and contralateral leg of all subjects of the study^6^. Mast cells are dispersed throughout adipose tissue and tend to be enriched in fibrotic areas of fat (Fig. S3); however, not all adipose tissue samples contain fibrotic areas, and hence we only counted mast cells in the non-fibrotic areas. Representative images of mast cell staining at baseline and after cold (cold treated leg) are shown in Fig. 2A,B, and the quantification of mast cell density is shown in Fig. 2C. Cold significantly increased the mast cell per adipocyte ratio by approximately 1.6-fold in the cold-treated and contralateral legs of lean subjects (Fig. 2C; cold: P < 0.01; contralateral: P < 0.01). As expected^33^, subjects with obesity had a higher baseline level of mast cells than lean subjects (Fig. 2C; P < 0.01), and mast cells did not increase in number in SC WAT of the cold treated or contralateral legs of subjects with obesity (Fig. 2C), consistent with the lack of change in mast cell tryptase gene expression noted above.

We have previously shown that cold stimulates mast cell degranulation and histamine release *in vitro* and that histamine stimulates adipocyte UCP1 expression^7^. Here, we noted a diffuse pattern of tryptase staining in the adipose tissue after cold, indicating mast cell degranulation (Fig. 2B). Therefore, we determined whether cold stimulates mast cell degranulation *in vivo* in response to cold. When mast cells degranulate the pattern of tryptase staining changes from cellular staining, usually in a circular pattern, to diffuse staining that is punctate and larger than unstimulated mast cells. Closer examination of the pattern of tryptase staining in SC WAT of the cold treated leg revealed tryptase staining of intact and degranulated mast cells. Images of tryptase staining taken at higher magnification are shown in Fig. 2D,E. We quantified the number of degranulated mast cells and observed that cold treatment stimulated mast cell degranulation in SC WAT from both the cold-treated and contralateral legs in lean subjects (Fig. 2F; cold: P < 0.0001; contralateral: P < 0.01). Cold also stimulated mast cell degranulation in SC WAT from both legs in subjects with obesity (Fig. 2F; cold: P < 0.0001; contralateral: P < 0.01), even though there was no increase in total mast cells (Fig. 2C).

### *In vitro* studies on mast cell degranulation

**Figure 3:**
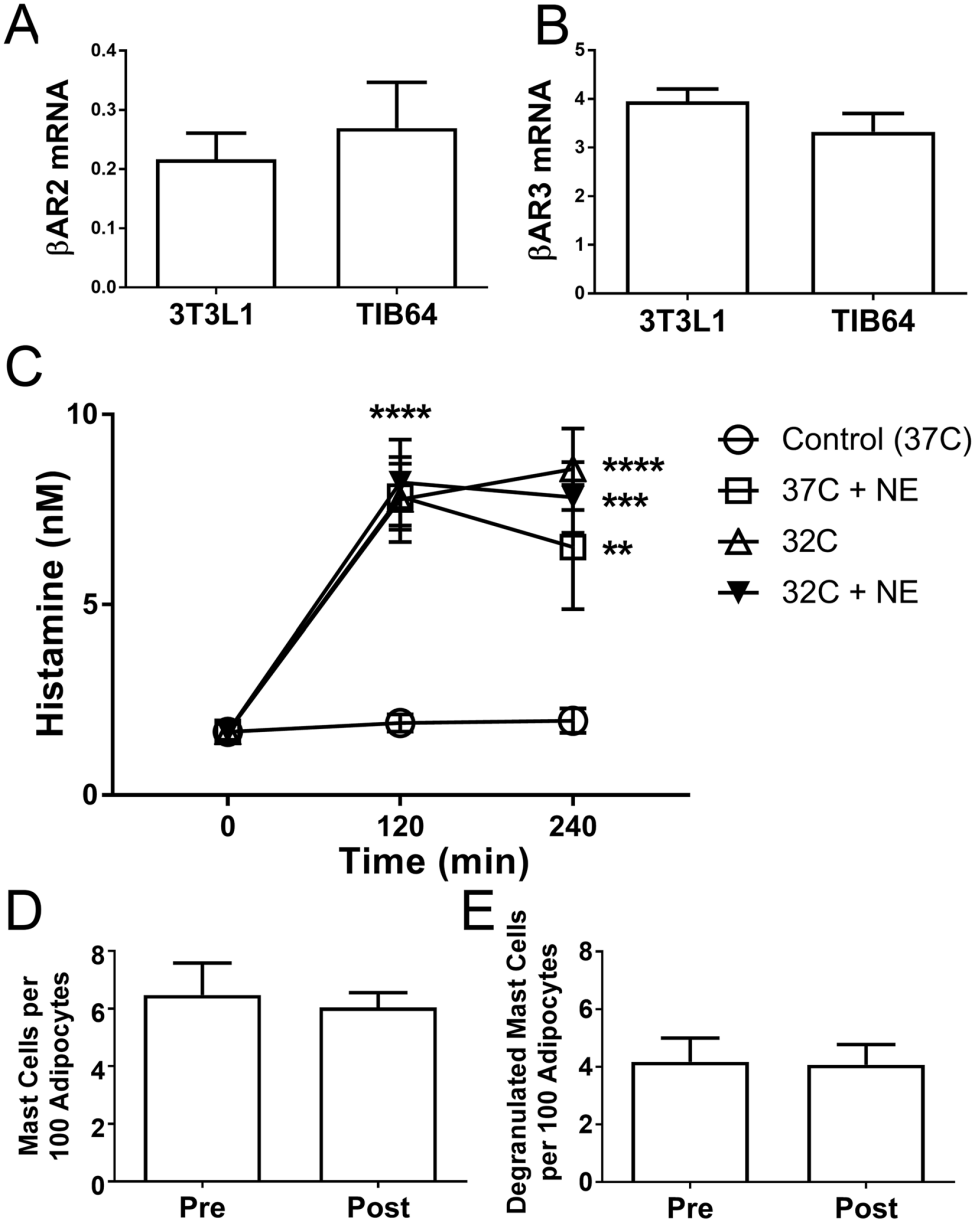
Mast cells express βARs and degranulate in response to norepinephrine. (**A**,**B**) βAR2 and 3 mRNA expression was determined in 3T3L1 and TIB64 mast cells by real-time RT PCR as described in methods. (**C**) TIB64 cells were changed into media at 32 C or 37 C with 0 or 100 nM norepinephrine (NE) for 0, 120 or 240 minutes as indicated. The media was removed from the cells and histamine determined as described in research design and methods. Data are presented as means ± SEM. The data were analyzed by ANOVA as described in research design and methods. **P < 0.01; ***P < 0.001; ****P < 0.0001. (**D**) Mast cell density and degranulated mast cells were quantified in SC WAT from human research participants with obesity treated with 50 mg mirabegron per day for 10 weeks. Data are presented as means ± SEM and were analyzed by a paired, two-tailed student’s t test (n = 6).

We previously found that cold causes mast cell degranulation *in vitro*, which possibly explains mast cell degranulation in the cold treated leg *in vivo*. However, the contralateral leg demonstrated equivalent mast cell degranulation (Fig. 2F), suggesting a different mechanism, and not solely a temperature effect. We previously found that the level of UCP1 and TMEM26 induction is the same in SC WAT of the contralateral leg as the cold treated leg, suggesting that cold stimulated the SNS, which then stimulated WAT beiging^6^. To determine whether mast cells could be responsive to SNS stimulation, we evaluated βAR expression on mast cells and determined whether NE stimulates mast cell degranulation. As shown in Fig. 3A,B, TIB64 mast cells express β2AR and β3ARs at similar levels as 3T3L1 adipocytes; we did not detect βAR1 expression in TIB64 cells. We treated TIB64 mast cells with norepinephrine (100 nM) at 32 °C and at 37 °C and measured histamine levels in the media 120 and 240 min after treatment. Norepinephine at 37 °C stimulated histamine release to the same extent as cold (32 °C) treatment; however, treatment with NE at 32 °C did not result in more histamine release than cold (32 °C) (Fig. 3C).

We previously showed that mirabegron, a specific β3AR agonist, stimulated pHSL phosphorylation and induced UCP1 and TMEM26 protein expression in SC WAT, suggesting βAR3 receptor stimulation *in vivo*^6^. Here, we determined whether mirabegron treatment stimulated mast cell degranulation in the same cohort of insulin resistant research participants with obesity (see^6^ for baseline characteristics). As shown in Fig. 3D,E, there was no change in mast cell number or degranulation in response to mirabegron, suggesting that cold-mediated SNS activation and not just β3-adrenergic receptor activation is necessary to cause mast cell degranulation *in vivo*.

## Conclusions

The results presented in this study suggest that mast cell degranulation contributes to the induction of UCP1 in SC WAT by cold. Mast cells express βARs, and degranulate and release histamine in response to norepinephrine, suggesting that they are responsive to SNS activation.

## Discussion

Results from gene expression analysis suggested that mast cells are positive regulators of SC WAT beiging, which occurs in response to acute cold^6^. We found that cold induced mast cell recruitment and degranulation in SC WAT of lean research participants and mast cell degranulation in research participants with obesity. These effects on mast cells occurred equally in SC WAT from both the cold treated and contralateral legs, and we furthermore demonstrated that norepinephrine stimulates mast cell degranulation *in vitro*. Together, these observations and our previous work implicating mast cells as positive regulators of seasonal beiging^7^, strongly support a role for mast cells as positive regulators of SC WAT beiging, adding them to the list of immune cells involved in this process^13^.

An important finding from our previous study was that SC WAT beiging and increased UCP1 protein occurred to the same extent in the contralateral leg as the cold treated leg, suggesting that cold activated the SNS^6^. Here, we found that mast cell degranulation is also stimulated to the same extent in SC WAT of the contralateral leg as the cold treated leg, suggesting that mast cell degranulation may be stimulated by SNS activation. *In vitro* studies demonstrated that mast cells express β-adrenergic receptors and that norepinephrine induces mast cell degranulation, providing a mechanistic link between cold and mast cell degranulation. Indeed, mast cells are found in in anatomic association with nerves in other tissues^34^, indicating that mast cells may be an important link between the nervous system and immune cells. The findings of this study, in addition to our previous study on seasonal SC WAT beiging in humans^7^, suggest that β-adrenergic stimulation and subsequent degranulation and histamine release are the mechanisms by which mast cells are involved in stimulation of adipose beiging.

Previously, we demonstrated that mast cells are cold sensors; mast cells exposed to a physiologic cold exposure (32 °C) degranulate, releasing histamine *in vitro*^7^. Adipocytes express histamine receptors, and histamine receptor activation increases cAMP, stimulating lipolysis and UCP1 expression in adipocytes^7^. Previous studies have shown that histamine promotes thermogenic responses by additional mechanisms besides these direct effects on adipocytes. Histamine increases blood flow in BAT, which is an important physiologic response for thermogenesis^35,36^, and it also renders cold sensitive nerves more sensitive to stimuli^37^. Overall, results from these studies^35–37^ and the current study demonstrating mast cell degranulation *in vivo* in response to cold and the responsiveness of mast cells to catecholamine suggest a positive role for histamine in promoting thermogenic responses to defend against cold. It is of interest to note that stimulation of histamine receptors in the brain positively affects energy expenditure and UCP1 induction in BAT in rodents^38–40^. Thus, several studies indicate that histamine acts at multiple sites to promote thermogenesis. However, one study in rodents indicates that mast cells have several negative effects in the context of obesity, including the suppression of UCP1 in BAT^33^. It is therefore possible that mast cells have different effects on beiging in SC WAT and BAT in humans and rodents.

We gained additional insight into SC WAT beiging from the analysis of gene expression in this study. Importantly, the multiple correlations analysis identified CCL26 as a gene whose change in expression positively correlated with the change in UCP1 expression in the cold treated leg. CCL26 is a chemokine for CCR3 expressing cells such as mast cells, basophils, and eosinophils^27–29^, and induction of CCL26 by acute cold exposure could thus be the mechanism leading to mast cell recruitment to adipose tissue. CCL26 was also identified as a significantly induced gene in the contralateral leg; therefore, CCL26 may be increased by SNS activity. CCL26 may thus be an important link between the SNS and the recruitment of immune cells involved in type 2 immune responses in adipose tissue. CCL26 mRNA was not increased in the cold treated leg. It is possible that cold caused a transient increase in CCL26. Further studies on the kinetics of CCL26 mRNA and protein induction will be necessary to elucidate the role of CCL26 in mast cell recruitment into adipose. We also found that the change in CIDEA gene expression inversely correlated with the change in UCP1 protein expression (Table 3). CIDEA increases with adipose beiging but is known to inhibit UCP1 activity in mice^41^. It will be import and to investigate this possible regulatory mechanism on UCP1 function in humans in future studies.

We found that VEGF and other genes that regulate vascularity were changed in the contralateral leg; in addition to being pro-angiogenic, VEGF also induces beiging^31,32^. Similarly, angiopoiten-2 was induced in both the cold treated and contralateral leg. Angiopoiten-2 was thought to be antiangiogenic; however, a recent study found that it is pro-angiogenic in adipose tissue^42^. One feature of beige adipose is increased vascularity to deliver oxygen and nutrients and remove heat, and we therefore measured capillary and vessel density. We did not find changes in SC WAT capillary density by histochemistry after acute cold; however, it may require longer than the 10 day time period of this study to increase capillarity. Finally, multiple correlations analysis identified angiopoietin like-4 (angptl4) as a gene negatively associated with UCP1. Angptl4 inhibits lipoprotein lipase (LPL), and it would thus be important to determine whether cold stimulates LPL to deliver more lipid to beige adipose for oxidation in future studies.

Finally, we note that a limitation of this study is that it was not adequately powered to detect an interaction between sex and mast cell recruitment or degranulation, which is an important question. In our previous study we were not able to detect an interaction between sex and adipose beiging from this same set of research participants^6^.

In conclusion, this study shows that mast cells are recruited to SC WAT of lean subjects in response to cold. Cold stimulates mast cell degranulation in all subjects regardless of baseline BMI, consistent with stimulation of SC WAT beiging^6^. This study, in combination with our recent study on the role of mast cells in SC WAT beiging in the winter, suggests that mast cells promote SC WAT beiging. Future clinical studies will employ histamine receptor antagonists or mast cell stabilizers to determine the importance of mast cells to SC WAT beiging *in vivo*.

## Supplementary information

Supplementary Information

Table S1

## Data Availability

The datasets generated during and/or analyzed during the current study are available from the corresponding author on reasonable request.
