# Supplementary material for: Adipose Tissue Mast Cells Promote Human Adipose Beiging in Response to Cold
Source: Sci Rep. 2019 Jun 17;9:8658. doi: 10.1038/s41598-019-45136-9 (PMC6572779; doi:10.1038/s41598-019-45136-9)
Supplement: Supplementary file 1 — Supplementary Information [file 41598_2019_45136_MOESM1_ESM.pdf]

Scientific Reports

Supplementary Information

Adipose Tissue Mast Cells Promote Human Adipose Beiging in Response to Cold

Brian S. Finlin, Amy L. Confides, Beibei Zhu, Mary C. Boulanger, Hasiyet Memetimin, Kyle W. Taylor, Zachary R. Johnson, Philip M. Westgate, Esther E. Dupont-Versteegden, and Philip A. Kern

Inventory of Supplementary Information

Supplementary Figure 1

Supplementary Figure 2

Supplementary Figure 3

Supplementary Table 2

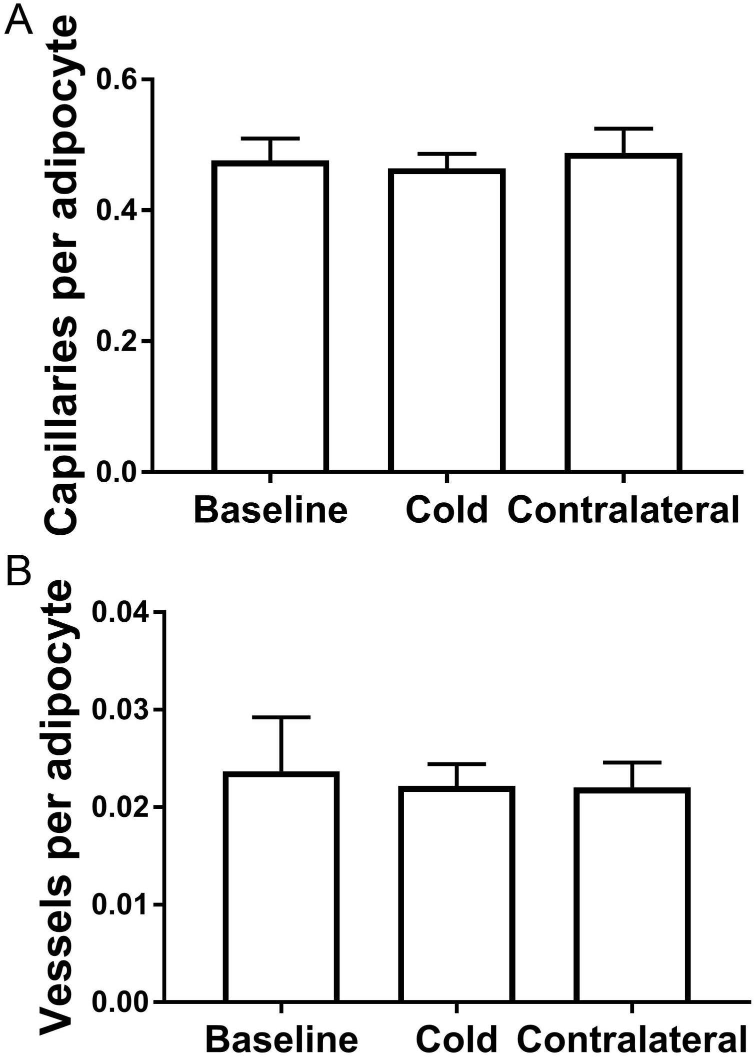

Figure S1. Capillary and vessel density in SC WAT of lean research participants in response to acute cold treatment. A) capillaries and B) vessels were quantified in SC WAT as described in methods. Data represent means ± SEM (n=6).

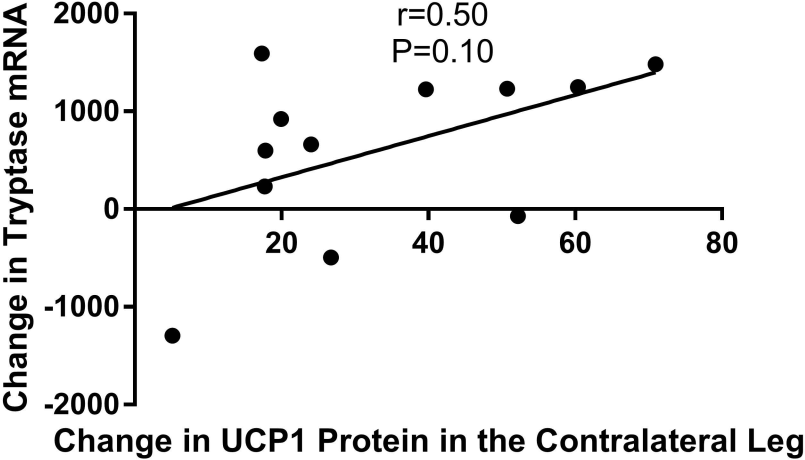

Figure S2. Correlations of changes in tryptase gene expression with changes in UCP1 in the contralateral leg. A) the change tryptase versus the change in UCP1 in the contralateral leg is shown. The data were analyzed by linear regression analysis, and Pearson correlation coefficients (r) and P values are shown (n=12).

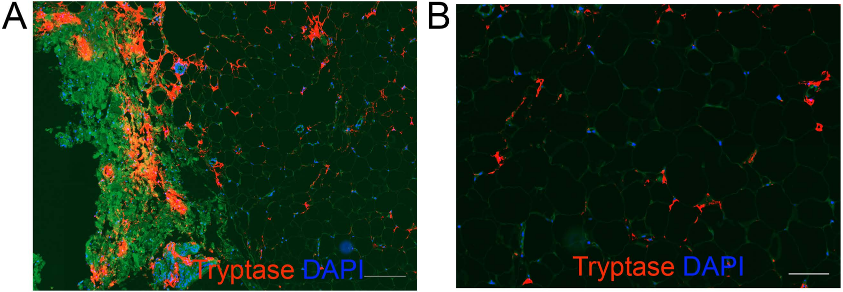

Mast cells in SC WAT   Mast cells among adipocytes

Figure S3. Mast cell staining in human SC WAT.

A) tryptase staining shows the location of mast cells in fibrotic areas, which are visualized by auto fluorescence in the FITC channel, and in fields of adipocytes (scale bar: 200 μm).

B) A higher magnification of a shows mast cells among adipocytes (scale bar: 100 μm).

Table S2. Primer sequences.

	Forward	Reverse
mADRB1	TCC CCC GGA GCT CCC TC	CTG TCC ACA GTG GTT GTC CC
mADRB2	GGG AAC GAC AGC GAC TTC TT	GCC AGG ACG ATA ACC GAC AT
mADRB3	GAC TAC AGA CCA TAA CCA ACG TG	CCT GGT GGC ATT ACG AGG A
mACTB F	GGC TGT ATT CCC CTC CAT CG	CCA GTT GGT AAC AAT GCC ATG T
mPPIA F	GAG CTG TTT GCA GAC AAA GTT C	CCC TGG CAC ATG AAT CCT GG
mPPIB F	GGC TCC GTC GTC TTC CTT TT	ACT CGT CCT ACA GAT TCA TCT CC
mTBP F	ACC GTG AAT CTT GGC TGT AAA C	GCA GCA AAT CGC TTG GGA TTA
mTUBB F	AGT AGA GCT CCC AGC AGG C	TCT CAC CCT CGC CTT CTA AC
mUBC F	GAC GTC CAA GGT GAT GGT CT	TCC AGA AAG AGT CCA CCC TG
